# Landscape analysis of NTD diagnostics and considerations on the development of a strategy for regulatory pathways

**DOI:** 10.1371/journal.pntd.0010597

**Published:** 2022-07-05

**Authors:** Hye Lynn Choi, Camilla Ducker, Susie Braniff, Daniel Argaw, Anthony W. Solomon, Bettina Borisch, Deusdedit Mubangizi

**Affiliations:** 1 World Health Organization, Geneva, Switzerland; 2 Institute of Global Health, University of Geneva, Geneva, Switzerland; Liverpool School of Tropical Medicine, UNITED KINGDOM

## Abstract

Access to quality-assured, accurate diagnostics is critical to ensure that the 2021–2030 neglected tropical disease (NTD) road map targets can be achieved. Currently, however, there is limited regulatory oversight and few quality assurance mechanisms for NTD diagnostic tools. In attempting to address such challenges and the changing environment in regulatory requirements for diagnostics, a landscape analysis was conducted, to better understand the availability of NTD diagnostics and inform future regulatory frameworks. The list of commercially available diagnostics was compiled from various sources, including WHO guidance, national guidelines for case detection and management, diagnostic target product profiles and the published literature. The inventory was analyzed according to diagnostic type, intended use, regulatory status, and risk classification. To estimate the global need and size of the market for each type of diagnostic, annual procurement data were collected from WHO, procurement agencies, NGOs and international organizations, where available and global disease prevalence. Expert interviews were also conducted to ensure a better understanding of how diagnostics are procured and used. Of 125 diagnostic tools included in this analysis, rapid diagnostic tools accounted for 33% of diagnostics used for NTDs and very few diagnostics had been subjected to regulatory assessment. The number of tests needed for each disease was less than 1 million units per annum, except in the case of two diseases, suggesting limited commercial value. Despite the nature of the market, and presumed insufficient return on commercial investment, acceptable levels of assurance on performance, quality and safety of diagnostics are still required. Priority actions include setting up an agile, interim, stepwise risk assessment mechanism, in particular for diagnostics of lower risk, in order to support national NTD programmes and their partners with the selection and procurement of the diagnostics needed to control, eliminate and eradicate NTDs.

## Author summary

Rapidly evolving landscape of in-vitro diagnostics is posing quality assurance challenges for manufacturers and regulators as well as creating product selection dilemmas for procurers, donors, and national programmes. In attempting to address such challenges, a landscape analysis of Neglected Tropical Diseases (NTD) diagnostics was conducted to provide insights for all stakeholders to support a future regulatory framework for NTD diagnostics. The inventory of commercially available diagnostics was compiled and analyzed and the global need and size of the market for each type of diagnostic was estimated. The results showed low commercial value and limited regulatory oversight on NTD diagnostics. Despite the insufficient return on commercial investment, reasonable assurance of product safety, performance and quality is required. One of the priorities is to establish a risk assessment mechanism, in particular for NTD diagnostics of lower risk, in order to support national NTD programmes, procurement agencies, donors and other partners with the selection and procurement of the diagnostics needed to achieve the 2021–2030 NTD road map targets.

## Introduction

Access to quality-assured and accurate diagnostics is key for treatment, control and prevention of infectious diseases [1]. Improved access to health products including diagnostics is an essential enabler for universal health coverage and is highlighted in targets 3.8 and 3.b of the Sustainable Development Goals 2030 [2, 3]. For neglected tropical diseases (NTDs), which disproportionately affect populations with poverty and low visibility [4], effective diagnostics are particularly critical in accelerating progress towards disease elimination and reducing morbidity and mortality.

The 2021–2030 NTD road map highlights crucial areas for action to ensure that public health targets for 2030 are achieved. One of these areas is diagnostics, which are needed for confirmation of disease, mapping, screening, surveillance, and monitoring and evaluation [5]. Accurate diagnostics can accelerate the progress of elimination programmes by correctly identifying infection, for example, in human African trypanosomiasis (HAT), leprosy and yaws [5]. Early detection through diagnostics reduces morbidity by reducing disease progression; this also reduces the accompanying financial burdens for patients and health systems. Accurate diagnosis also helps national NTD programmes make appropriate decisions on intervention for (for example) the frequency and duration of mass drug administration.

Despite the critical role of diagnostics in achieving NTD targets, a number of current challenges can be identified. For some NTDs, inadequate diagnostic tools are available or no appropriate diagnostics exist at all [5]. Having limited availability of tests of high sensitivity and specificity delays disease elimination if all true cases need to be identified and treated. While laboratory testing, including microscopy, plays a central role in the diagnosis of many NTDs, low- and middle-income countries often lack an integrated network of laboratories, equipment and trained staff [6]. The lack of a commercially viable market for NTD diagnostics is another major barrier, with the economic aspect further hampered by scarce public investments and an absence of coordinated global need estimates and procurement mechanisms [7].

Recognizing the challenges that NTD programmes face and the essential role that diagnostic tools play in reaching the 2030 targets, the Diagnostics Technical Advisory Group (DTAG) was established in 2019 to incubate the collaborative development of new diagnostic tools and to provide strategic advice to WHO and its partners [8]. Following a prioritization exercise to identify the most urgent needs, a number of target product profiles (TPPs) were developed through DTAG’s disease-specific subgroups and posted on WHO’s website for public consultations. The DTAG also recommended the establishment of cross-cutting subgroups to address issues that are common across diseases, including surveillance platforms, clinical diagnosis, microscopy and imaging, and manufacturing and regulatory pathway [9]; these sub-groups were established in 2021.

The manufacturing and regulatory pathways subgroup has been tasked by the DTAG to develop recommendations on standardizing the procedure for laboratory and field validation of new diagnostic tools, review regulatory processes for diagnostics tools used for population-based and individual testing, and provide recommendations on how to ensure country access to affordable, quality-assured diagnostics. In its first meeting, held in May 2021, the subgroup considered the potential impact on NTD diagnostics of the European Union’s in vitro diagnostic medical devices regulation (EU) 2017/746 (IVDR) [10], a new regulatory framework. IVDR aims to establish a more rigorous, standardized and better-regulated market for diagnostics and medical devices within the European Union. With IVDR rollout, expected in the next six years [11], it is anticipated that some diagnostics currently on the market might become temporarily unavailable or be permanently discontinued [12]. The consequences of IVDR implementation for NTD diagnostics are even more unclear, in part because the market serves the poorest of the poor, is small, fragmented and largely invisible. Serious concerns were expressed by subgroup members about manufacturers withdrawing products from the marketplace if anticipated returns do not justify the resources needed for investment. On the other hand, NTD diagnostics are used in many countries where there are limited or no financial resources and technical expertise to regulate the market for medical devices; and a lack of regulatory oversight and inadequate quality assurance mechanisms for diagnostics could seriously hamper progress towards 2030 targets. Therefore, keeping a balance between ensuring quality and safety of NTD diagnostic tools and maintaining or expanding manufacturers’ interest in this space is going to be both important and somewhat fraught.

Contemporary financial, technical and political challenges that the NTD community faces as well as these and other changes in regulatory environments prompted the current attempt to conduct a landscape analysis, so as to better understand the current availability, intended uses, potential risks, regulatory status, and estimated market size of NTD diagnostics. Through this landscape analysis we aim to provide insights for manufacturers, partners, procurers and national NTD programmes to support a future regulatory framework for NTD diagnostics.

## Method

The inventory of diagnostics used for NTD programmes for selected NTDs (setting aside those that currently have no commercially-available diagnostic tests), was compiled from multiple sources. These comprised: available WHO diagnosis and treatment guidance for each disease or disease group, reports from WHO technical advisory subgroups, the WHO technical report series, national guidelines for NTD case detection and management, diagnostic target product profiles published by WHO, and the published literature [13–31]. For the inventory, the definition of an in-vitro diagnostic (IVD) medical device was adopted from WHO, namely that it is a medical device, used alone or in combination, intended by the manufacturer for the examination of specimens to provide information on diagnostic, monitoring or compatibility purposes [32]. We only included IVDs and microscopy kits currently available in the inventory and did not consider tests either currently being developed or discontinued for marketing.

NTD diagnostics were grouped into the four most commonly used diagnostic types–immunochromatographic or rapid diagnostic tests (RDT); enzyme immunoassays (EIA), including chemiluminescence immunoanalysers; nucleic acid tests (NAT), including polymerase chain reaction- (PCR) and loop-mediated isothermal amplification-based assays; microscopy kits; and other diagnostics such as agglutination tests, trypanolysis tests and confirmatory assays. The intended use of individual diagnostic tools was extracted from the product label, when available, and WHO programme guidance or strategy documents and then mapped for the diagnostic strategy of each disease as outlined in the 2021–2030 NTD road map [5].

Risk classification of diagnostics is an important parameter in determining the depth of assessment appropriate to a given assay. It considers the risk posed by the product to public health and individuals, and the risk of an incorrect result arising from the use of a given diagnostic in specific settings [33]. For this analysis, we adopted the risk classification proposed by the International Medical Device Regulators Forum in 2021 and outlined in its Principles of IVD Medical Devices Classification [34] (Table 1). For risk classification, we considered the intended use of diagnostics in the context of NTD programmes’ goals as well as the indications for use specified by the manufacturer. The importance of the information obtained from the use of diagnostic to the overall diagnosis process—whether the result of the diagnostic is the sole determinant for decision-making or one of several determinants such as clinical exams—was based on the algorithms for the diagnosis of each disease recommended in current WHO guidance. This process involved consultations with WHO disease leads and regulatory experts for IVDs.

**Table 1:** Risk classification system for IVDs.

Class	Risk level
A	Low Individual Risk and Low Public Health Risk
B	Moderate Individual Risk and Low Public Health Risk
C	High Individual Risk and/or Moderate Public Health Risk
D	High Individual Risk and High Public Health Risk

To collect information on the regulatory status of each diagnostic, the list of diagnostics cleared or approved by stringent regulatory authorities was searched. These included the US FDA and Health Canada [21], complementing the information included on the product label. Products with CE (Conformité Européenne) marks were also listed.

For the estimated size of the market for each type of diagnostic for each disease, we collected annual procurement volumes as established by WHO, procurement agencies, NGOs and international organizations. Where there were no procurement data available, we estimated the market size based on the number of cases of the disease or disease group reported to WHO per year and the recommended diagnostic test for screening and individual treatment. For diagnostics used for mapping, in decisions to start or stop mass drug administration, or in surveillance, we estimated demand based on the number of tests conducted in countries having implemented these activities in the past three years. We also conducted interviews with experts in the field to gain a better understanding of how diagnostics were procured in countries that use domestic funds.

## Result

**Fig 1:**
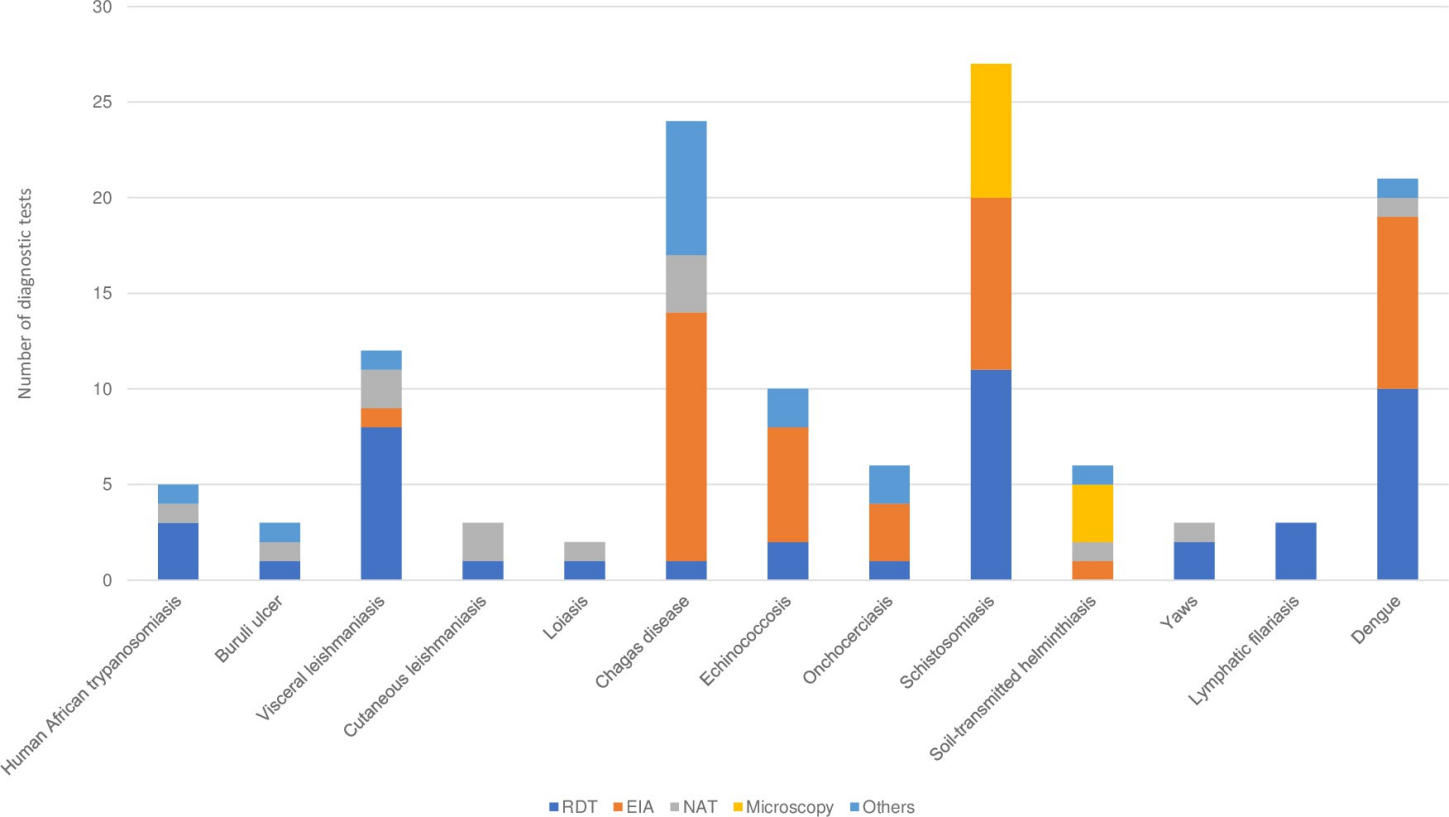
Number and type of diagnostics available for NTDs. (A) RDT, Rapid diagnostic tests; EIA, enzyme immunoassay including chemiluminescence immunoanalysers; NAT, nucleic acid test including polymerase chain reaction and loop-mediated isothermal amplification. (B)The result includes diagnostics that are used for more than one disease (for example, microscopy tests for schistosomiasis and soil-transmitted helminths).

The number of diagnostics commercially available for NTDs per type of diagnostic is presented in Fig 1. Of 125 diagnostics tools included in this analysis, RDTs account for 33% of diagnostics used for NTDs, although there are very few or no RDT available for cutaneous leishmaniasis, loiasis, onchocerciasis and soil-transmitted helminths. The number of diagnostics available for each NTD was less than 10 (except in the case of dengue, Chagas and schistosomiasis).

For diseases requiring individual diagnosis for treatment (Table 2), the intended use of the diagnostics has been mapped to key components of NTD programmes, from confirmation of disease to screening and surveillance.

**Table 2:** Number of diagnostics per purpose of diagnostics/intended use.

Disease	Screening	Confirm diagnosis	Surveillance
Human African trypanosomiasis	2	3	1
Buruli ulcer	0	3	0
Visceral leishmaniasis	0	12	0
Cutaneous leishmaniasis	0	3	0
Loiasis	0	1	1
Chagas disease	12	15	0
Dengue	0	11	10
Yaws	1	1	3
Echinococcosis	0	3	0

(A) Some diagnostics were counted more than once, when serving multiple purposes. For example, Bioline HAT used for both screening and case management is counted twice.

For diseases recommended to be controlled in part by mass drug administration, diagnostics may be used to guide decisions for mapping, initiating and stopping mass drug administration, surveillance or verifying the interruption of transmission (Table 3).

**Table 3:** Number of diagnostics per purpose of diagnostic/intended use.

Disease	MDA decision/mapping	Post intervention/surveillance
Onchocerciasis	6	0
Schistosomiasis	9	9
Soil-transmitted helminthiasis	4	1
Lymphatic filariasis	3	0
Yaws	1	3

Risk classification for diagnostics included in this analysis is presented in Table 4. For diseases such as lymphatic filariasis, soil-transmitted helminthiasis, schistosomiasis, onchocerciasis and yaws, national elimination and control activities are implemented where preventive chemotherapy, i.e. regular administration of medicines to all population groups at risk of morbidity [35], is the main tool. Medicines used for mass drug administration, such as albendazole, mebendazole, ivermectin, praziquantel and azithromycin, have well documented safety profiles with only mild adverse events and low risk for individuals treated [36]; the associated diagnostics are therefore assigned to risk class B.

**Table 4:** Risk classification.

Risk classification	Diagnostics
A	None
B	Lymphatic filariasis RDT, yaws RDT, yaws NAT, soil-transmitted helminth microscopy kits, schistosomiasis RDT, schistosomiasis EIA, schistosoma microscopy kits, onchocerciasis RDT, onchocerciasis EIA, loiasis RDT
C	Dengue RDT, dengue EIA, echinococcosis RDT, echinococcosis EIA, human African trypanosomiasis RDT, human African Trypanosomiasis NAT, visceral leishmaniasis, cutaneous leishmaniasis, loiasis NAT, Buruli ulcer RDT, Buruli ulcer NAT
D	Chagas disease RDT, Chagas disease NAT

(A) RDT, Rapid diagnostic tests; EIA, enzyme immunoassay; NAT, nucleic acid test. (B) This table does not include diagnostics that were excluded from this analysis (i.e. scabies, leprosy, trachoma).

Diagnostics used to determine individual disease status, where there is a risk that an erroneous result might lead to a patient management decision that threaten life or livelihood (e.g. human African trypanosomiasis, visceral leishmaniasis, cutaneous leishmaniasis, loiasis, echinococcosis, Buruli ulcer), were placed in risk class C.

For Chagas disease, a potentially life-threatening illness, where blood screening is critical to prevent infection through transfusion and organ transplantation or congenital transmission, diagnostic tools were classified as risk class D.

The extent to which NTD diagnostics have been subject to regulatory assessment was very limited. Analysis indicated that, of 125 diagnostics, only a few were either CE marked (18) or approved or cleared by a stringent regulatory authority (4). Diagnostics cleared by US FDA were Inbios Kalazar Detect, CL Detect Rapid test, SMART Leish and Chagas Detect plus rapid test. This contrasts with diagnostics for malaria and HIV/AIDS, for which 19 of 23 RDTs for malaria and all 49 HIV diagnostics procured by the Global Fund are either WHO-prequalified or stringently assessed by regulatory authorities of the founding members of the Global Harmonization Task Force [37].

**Fig 2:**
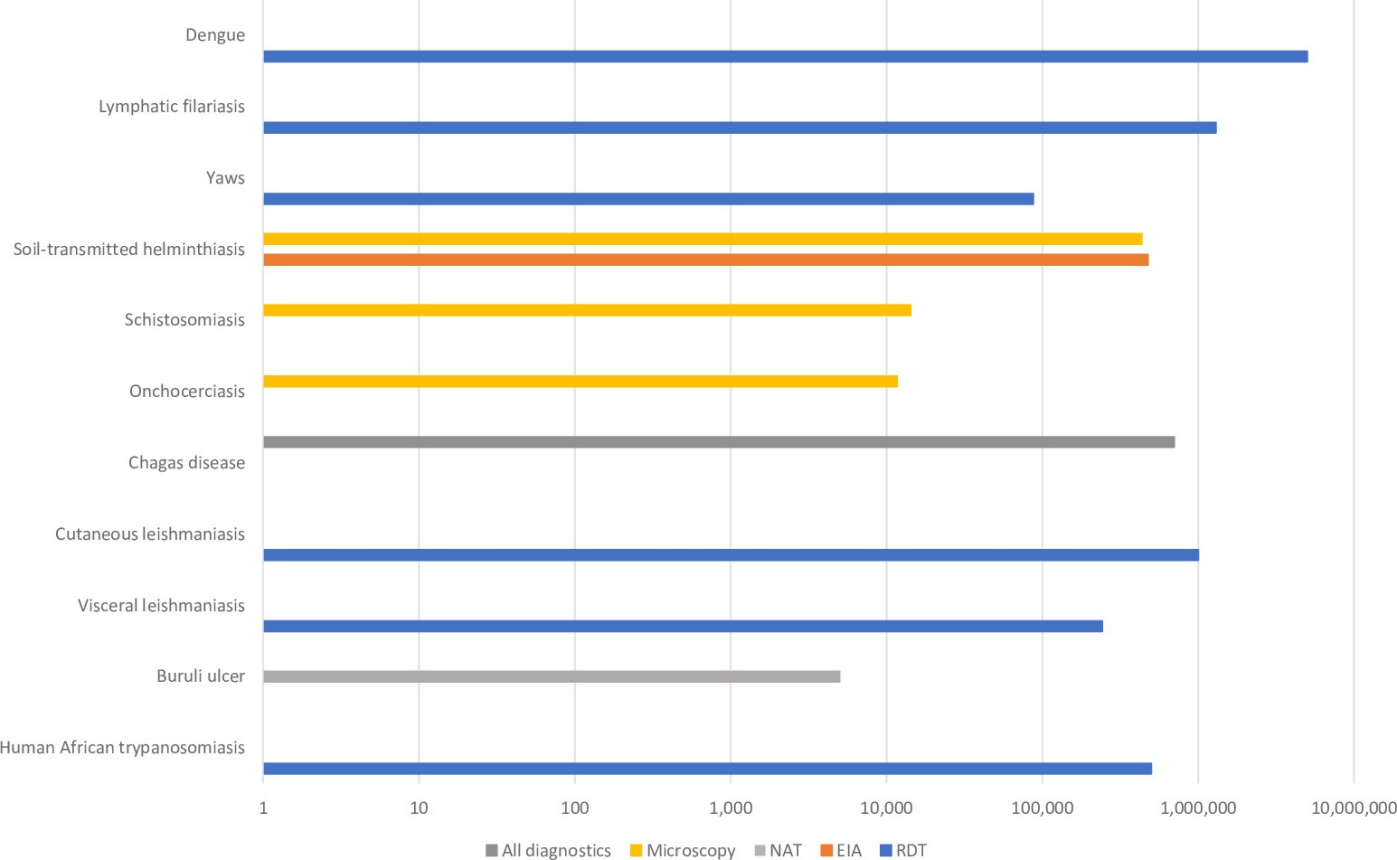
Estimated global need and market size for NTD diagnostics. (A) Volume of diagnostics for yaws, lymphatic filariasis, soil transmitted helminthiasis, visceral leishmaniasis, Buruli ulcer and human African trypanosomiasis (HAT) is based on median procurement volume for 2017–2019. (B) Volume of diagnostics for schistosomiasis and onchocerciasis is based on WHO Preventive Chemotherapy Epidemiological Data Reporting Form reported from endemic countries from 2017–2019. (C) For Dengue, Chagas, cutaneous leishmaniasis, for which procurement volumes were not available, market size was estimated based on global disease prevalence. For Chagas disease, the estimated volume is for any type of diagnostic.

Estimated market size for NTD diagnostics, based on historical procurement volume or commonly used diagnosis practice, accounts for the diverse landscape in Fig 2. Except for dengue and lymphatic filariasis, the number of tests needed for each disease was considerably less than 1 million units per annum, between 5,000 to 700,000 units for each disease or disease group. RDTs accounted for 90% of the total NTD market size.

## Discussion

This landscape analysis presents the diversity and complexity of diagnostic tools currently being employed by NTD programmes. For diseases and disease groups with declining prevalence and incidence rates, NTD programmes may face new challenges in achieving 2030 targets, while surveillance is a critical requirement in validating the elimination or eradication of diseases. Led by DTAG and its subgroups, several TPPs were recently developed to guide the development of new diagnostics that can address current gaps [8, 38].

Although several diagnostic options may be available for each disease, many of these have limitations in quality, safety and performance. In case of microscopy kits, the methodology has fewer quality, safety and performance concerns but is frequently time-consuming and requires the training of large number of skilled personnel. As confirmed through our analysis, the lack of a defined structure for regulatory clearance or approval for NTD diagnostics has translated to limited regulatory oversight by stringent authorities to date. This is further compounded by the lack of regulatory systems for medical devices in many low- and middle-countries as a result of limited financial resources and shortfalls in the technical expertise needed to transition from an unregulated market to a comprehensive medical devices framework [39]. In our attempt to guide and assist NTD programmes in the selection and procurement of available NTD diagnostics, we propose a progressive or stepwise approach to relevant regulatory pathways.

### Stringent regulatory assessment

Assessments of IVDs performed by the WHO Prequalification programme or founding members of the Global Harmonization Task Force (GHTF) (now replaced by the International Medical Device Regulators Forum (IMDRF)) ensures an additional quality assessment over and above the nationally-led or programme-led product evaluation undertaken for product selection or procurement. Reference jurisdictions include the European Union, the United States Food and Drug Administration, Health Canada, the Australian Therapeutic Goods Administration and Japan’s Ministry of Health, Labour and Welfare.

WHO Prequalification of IVDs provides a comprehensive quality assessment of individual, commercially available tests through review of a full product dossier, performance evaluation, manufacturing site inspection and labelling review. Once prequalified, the IVD is included in the WHO List of Prequalified In Vitro Diagnostic Products and its relevant information becomes available in the WHO website, subject to the protection of commercially-sensitive confidential information. Publicly available content includes the names of products and of manufacturers that applied for prequalification, a WHO public assessment report, a WHO public inspection report and any negative outcome of the assessment such as product alerts [40]. The manufacturer submitting a dossier for Prequalification is charged a fee, to partially cover the cost of the assessment. Although no NTD diagnostics are currently eligible for submission to this process, WHO Prequalification plans to include a few selected NTD diagnostics in its scope following stakeholder consultation and with the concurrence of the Strategic Advisory Group of Experts on In Vitro Diagnostics [41]. A potential benefit of prequalification is accelerated registration of IVDs by National Regulatory Authorities through collaborative registration procedures, in which confidential prequalification assessment reports are shared with participating regulatory authorities to avoid repetitive assessments [42].

Our landscape analysis of NTD diagnostic tools indicates that some diagnostics used for echinococcosis, human African trypanosomiasis, visceral leishmaniasis, cutaneous leishmaniasis, loiasis and Buruli ulcer have Class C or Class D status, implying potentially high risks to individuals. Stringent regulatory assessment of such NTD diagnostics will provide quality assurance for the benefit of all stakeholders, as we move closer to achieving eradiation, elimination and control targets.

### Risk-based assessment

For products that are neither prequalified nor have undergone stringent regulatory authority assessment, users or national NTD programmes may value a mechanism to provide interim evaluation of the risks and benefits associated with the given IVDs. This could, for example, support procurement decisions on products about which there is limited current understanding of product quality. WHO has established a mechanism, the Expert Review Panel for Diagnostics (ERPD), to help procurers and national programmes assess such risk and make informed decisions on a time-limited basis, usually one year [21]. The ERPD is integrated into the Quality Assurance policy for Global Fund and UNITAID procurement of diagnostics for HIV/AIDS, tuberculosis and malaria. The ERPD mechanism helps to accelerate access to diagnostic tools for populations in need if the associated risks are deemed to be less than the potential benefits. For NTD diagnostics in Class B, which implies limited risk to individuals, or new diagnostic tools becoming available, risk-based assessment, a less resource-intensive and interim measure, may be adequate to support programmatic decision-making. It is anticipated that some diagnostics assessed by ERPD will progress towards more stringent regulatory assessment. While some NTD diagnostics in higher risk classes may progress into WHO Prequalification following ERPD, many NTD diagnostics with lower risk do not have a similar independent quality assurance mechanism available. This poses financial, reputational and programmatic risks for procurers, donors and national disease control programmes in the use of such diagnostics. The proposed risk-based assessment for lower risk NTD diagnostics may require financial resources to set up the mechanism, develop tools and carry out the assessments for individual diagnostic test.

Our study is mainly limited by the availability of procurement data. The market size presented may be underestimated due to lack of information on procurement from national NTD programmes. Even if most countries are still relying on external support to secure access to NTD diagnostics, an increasing number of countries are using domestic funds to procure health products including diagnostics. Relevant information on this part of the market was not available for this analysis. Furthermore, demand for diagnostics may increase as national NTD programmes plan to implement surveys for mapping, prepare to take decisions to start or stop mass drug administration, seek data to support claims to verify elimination or conduct post-elimination surveillance studies. Another area where further research is needed is how to ensure quality of non-commercial diagnostics such as in-house diagnostics or home brew tests and diagnostics that were excluded in this analysis (i.e. scabies), as these tools are widely used for several NTDs and not under regulatory oversight.

We have provided herein a snapshot of the portfolio of NTD diagnostics currently on the market. The picture is not complete, but our attempt provides some insights into the NTD diagnostics landscape, its roles, regulatory gaps and the estimated demand for diagnostic tools. The diversity of diagnostics and their various uses in support of 2030 targets requires acceptable levels of assurance on performance, quality and safety, even though the diagnostics market is small. An agile, interim risk assessment mechanism is needed for NTD diagnostics in order to assess risks and potential benefits of candidate tests. This will help national NTD programmes, and their partners make informed decisions about the diagnostics needed to control, eliminate and eradiate NTDs.
